# Cultivating the Physician-Scientist Pipeline in Translational Aging: Early Outcomes from NIA StARR

**DOI:** 10.1093/geroni/igaf122.4403

**Published:** 2025-12-31

**Authors:** Alison Huang, Louis Nguyen, Heather Whitson, Ashley Price, Hannah Puttre, Zara Cooper, Anthony Viera, Christine Ritchie

**Affiliations:** University of California, San Francisco, San Francisco, California, United States; Brigham and Women’s Hospital, Boston, Massachusetts, United States; Duke University, Durham, North Carolina, United States; Duke University School of Medicine, Durham, North Carolina, United States; Massachusetts General Hospital, Boston, Massachusetts, United States; Brigham and Women’s Hospital, Boston, Massachusetts, United States; Duke University School of Medicine, Durham, North Carolina, United States; Massachusetts General Hospital, Boston, Massachusetts, United States

## Abstract

Despite efforts to increase the clinician-scientist pipeline, fewer physicians or surgeons are pursuing scientific careers, particularly in aging, threatening future advances in care. One critical challenge for early-stage physician/surgeon scientists is the demanding and inflexible nature of medical training, particularly residency training that offers few opportunities for sustained research engagement. The National Institutes of Health’s Stimulating Access to Research in Residency (StARR) R38 initiative represents a recent effort to directly integrate intensive, 1-2 year-long research experiences into residency. We present early insights from all StARR programs funded by the National Institute on Aging--the University of California San Francisco, Duke University, Brigham and Women’s Hospital, and Massachusetts General Hospital--which have collectively engaged 27 physicians/surgeons-in-training in basic, clinical, or translational aging science since 2021. Of the 15 NIA StARR graduates who have already completed residency, including 5 from traditionally underrepresented backgrounds, all have so far continued in academic training or careers, and 9 remain engaged in aging science. Mixed-methods evaluation data from graduating scholars demonstrate StARR’s meaningful impact on career trajectories and research commitment. However, program implementation has revealed key educational challenges: establishing effective interdisciplinary mentorship teams; balancing rigorous methodological training with productivity pressures; identifying sustainable funding pathways; and defining aging research focus across diverse specialties. These findings inform broader efforts to restructure medical education for research integration; while StARR’s early success suggests that systematic, extended research training during residency can effectively cultivate physician-scientists in aging, programmatic innovations are needed to address identified training barriers and ensure scalability across institutions.

